# Fused Filament Fabrication of Bio-Based Polyether-Block-Amide Polymers (PEBAX) and Their Related Properties

**DOI:** 10.3390/polym14235092

**Published:** 2022-11-23

**Authors:** Matthias Schär, Lucian Zweifel, Delal Arslan, Stefan Grieder, Christoph Maurer, Christian Brauner

**Affiliations:** Institute of Polymer Engineering, FHNW University of Applied Sciences and Arts Northwestern Switzerland, Klosterzelgstrasse 2, 5210 Windisch, Switzerland

**Keywords:** fused filament fabrication, bio-based, Pebax, Polyetherblockamid, TPE, 3D printing, additive manufacturing

## Abstract

This paper describes the application of poly(ether-block-amide) polymers, so-called Pebax, in fused filament fabrication (FFF). Pebax^®^ is a thermoplastic elastomer (TPE), a copolymer based on rigid polyamide and soft polyether blocks. By variation of the blocks, unique properties such as soft or rigid behaviour are tailored without additional additives and plasticisers. Pebax^®^Rnew^®^ polyamide blocks are bio-based and made from castor beans that allow the design of sustainable applications. In this study, two types of Pebax were selected, processing parameters were characterised, filaments were extruded and applied to FFF printing, and the final mechanical characteristics were determined. Both types were suitable for FFF processing with improved process stability due to less shear thinning and good mechanical performance. The connection strength between the grades was also described in the design context for complex parts with tailored soft or hard regions. Combining the two materials in one design is a promising concept, and the adhesion strength is close to the strength in the Z-direction of the flexible Pebax^®^Rnew^®^35R53 grade.

## 1. Introduction

Today’s industry anticipates emerging technologies capable of replacing conventional manufacturing processes with more economical and efficient production [1]. Additive manufacturing (AM) is one of these technologies, significantly reducing material waste and simplifying production processes, and on-demand production improves supply chain flexibility because the final product is manufactured close to the end-user [1,2]. AM has significantly advanced in recent years—from an immersive concept to a practical reality [3]. The increasing interest from research and industry reflects the potential and broad awareness of the technologies’ capabilities, which underlines how fast AM has evolved [3]. Today, applications of AM have been established in almost every field of day-to-day life, i.e., from printing continuous carbon fibre-reinforced composites [4], such as structural parts such as turbine blades [5] to orthopaedic modelling (bone surfaces) in biomedical applications [6]. Looking forward, many advanced manufacturing and mobility companies will see a vast increase in their industry’s use of AM by 2023. The forecast predicts a significant rise in using AM from 18% to 59% in aerospace, 22% to 53% in the chemical sector, and 17% to 44% in automotive. Hence, the automotive industry showed the slowest rate of adoption based on the presented forecast [7].

In conventional AM for polymer materials, parts are produced by building up individual layers that are joined together. The most common processes include fused filament fabrication (FFF) (also called fused deposition modelling (FDM)), stereolithography (SLA), multi-jet fusion (MJF), and selective laser sintering (SLS) [1]. The FFF process uses a heated nozzle that ejects molten polymer, depositing it in thin strands, one on top of another on a print bed, forming the 3D-printed part. A particular geometry is established with a moving nozzle or print bed (or both), whereby the polymer is extruded. FFF shows various advantages (large selection of materials, multi-material application, efficiency with no scrap material, etc.) but also certain disadvantages (high anisotropy due to low interlayer strength, etc.).

AM has been recognised as a sustainable and efficient technology. It allows manufacturers to use only the necessary amount of materials, a benefit that can add economic value by reducing both material and production costs [8]. The possibility of using a broader range of thermoplastic filament materials for FFF is of great importance [1,9]. Therefore, continuous effort is put towards developing polymers with more sustainable properties (bio-based, biodegradable, etc.). A positive aspect of, i.e., bio-based polymer materials is that CO_2_ is already removed from the atmosphere during the plant’s growth cycle. Here, thermoplastic filaments such as polylactic acid (PLA) have garnered much interest due to their low environmental impact [8]. This study uses bio-based poly(ether-block-amide) block copolymers (Pebax) with the tradename Pebax^®^Rnew^®^. The base material is beans of the castor plant. These plants are very frugal and thrive on unusable soil for conventional agricultural plants [10]. The castor beans are first crushed, and their oil is pressed for plastic production. Vegetable oils are excellent raw materials for polymer production, as they have an ideal chemical basis [11]. Bio-based polymer precursors are relatively easy to produce due to the active chemical building blocks with double bonds and esters [11,12]. The castor oil is converted into amino-11 monomers via several refinery steps. The polymer is then prepared by polycondensation of carboxylic acid polyamides (PA6, PA11, PA12) with an alcohol-terminated polyether, such as polytetramethylene glycol (PTMG) or polyethylene glycol (PEG).

**Figure 1:**
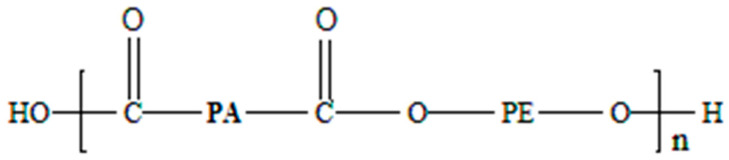
Chemical structure of poly(ether-block-amide) polymers (Pebax).

Pebax^®^ is an AB block copolymer with two types of segments. The structure is a linear chain in a systematic pattern and consists of flexible polyether and rigid polyamide (PA) blocks (Figure 1). The combination of rigid polyamide segments with flexible polyether segments results in copolymer blocks with a microphase-separated morphology [13]. This behaviour is caused by the polarity difference between the crystalline polyamide and amorphous polyether phases [13,14].

The properties of Pebax are individually adjustable depending on the proportion of polyamide and polyether. The crystalline polyamide block contributes to the polymer’s thermoplastic behaviour, whereas the amorphous polyether block causes the polymer’s elasticity and flexibility (see Figure 1). The melting temperature of Pebax block copolymers is mainly influenced by the polyamide block’s average molecular weight and the copolymer’s polyamide content. The melting range of the polyamide block and, thus, of the Pebax plastic is between 130 and 200 °C. The polyether block crystallises only between −30 °C and 20 °C. The glass transition temperature (*T_g_*) is about −50 °C [15]. 

The market for flexible FFF printing filaments offers a wide range of different materials. Table 1 compares the Pebax^®^Rnew^®^35R53 grades with some FFF printing filaments available on the market that have similar mechanical properties. However, with a Shore hardness of 25D, Pebax^®^Rnew^®^35R53 is one of the softest materials. In addition, Pebax^®^Rnew^®^35R53 filament has the lowest density of the listed materials. The modulus of elasticity of Pebax^®^Rnew^®^35R53 is slightly higher than the filaments with the same Shore hardness. However, determining the modulus of elasticity of flexible materials is complex, and different test standards have been used. Consequently, the modulus of elasticity values for comparing the materials are less meaningful than the Shore hardness, i.e., the material Kimya TPU-92A with a Shore hardness of 40 D has a significantly higher modulus of elasticity than the other materials.

This study reports the characterisation of the processing parameters relevant for FFF printing through differential scanning calorimetry (DSC), thermo-gravimetric analysis (TGA), and rheology. In this study, two Pebax^®^Rnew grades (Pebax^®^Rnew^®^35R53, Pebax^®^Rnew^®^1100) have been selected as the most promising candidates for extrusion and FFF processing. A filament from both grades was extruded, whereby the parameters were experimentally determined and the quality visually observed. Then, the filament was used for FFF printing, whereby optimal printing parameters were found, and mechanical properties from tensile test specimens were determined. Furthermore, multi-material tensile test specimens were printed to evaluate the adhesion strength between the two grades.

## 2. Material Characterisation

The study focused on the two grades, Pebax^®^Rnew^®^35R53 and Pebax^®^Rnew^®^1100, which both show very different mechanical properties—from highly flexible to rigid. Table 2 compares relevant properties for both grades used in the study.

### 2.1. Analysis of Glass Transition Temperature with Differential Scanning Calorimetry (DSC)

**Figure 2:**
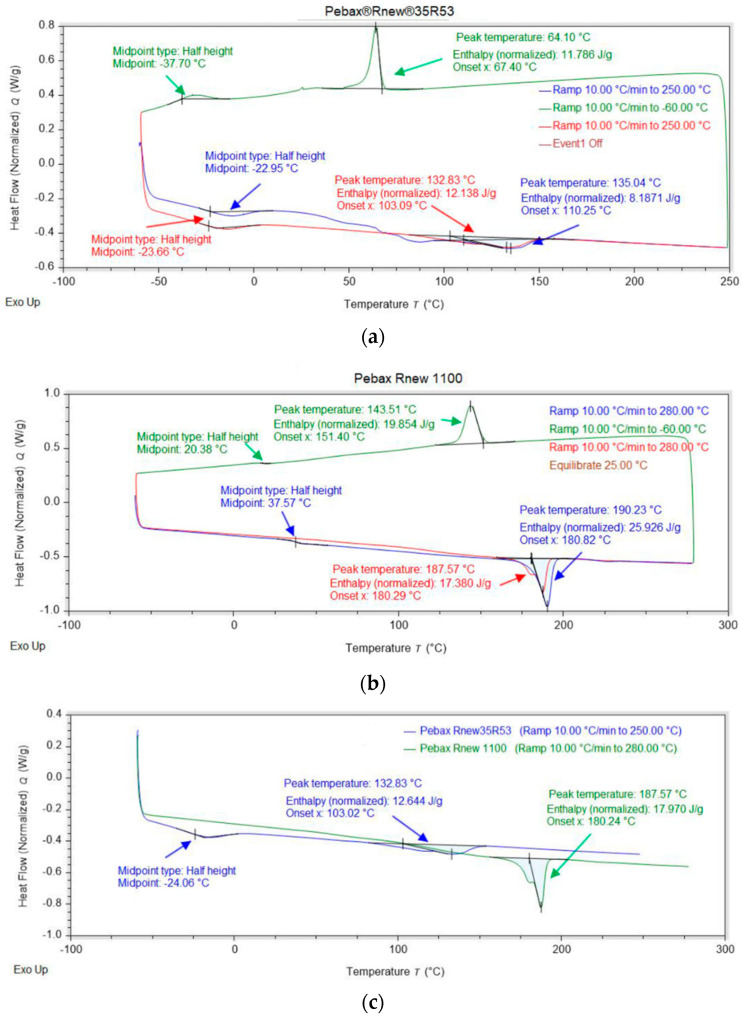
Dynamic differential scanning calorimetry (DSC) curves: blue, first heating; red, second heating; (**a**) Pebax^®^Rnew^®^35R53; (**b**) Pebax^®^Rnew^®^1100; (**c**) comparison of the second heating ramp of both grades.

The Pebax grades were analysed via differential scanning calorimetry (DSC) using a DSC 25 from TA instruments (New Castle, DE, USA). For the measurement, granules were used, which were then processed into filaments. Here, three samples with weights around 8 mg were heated (blue line) at a constant heat rate of 10 °C/min from −60 °C to 250 °C (Pebax^®^Rnew^®^35R53) respectively to 280 °C (Pebax^®^Rnew^®^1100). The same sample repeated the heating ramp once (red line). Figure 2 shows the derived heat flow over the temperature range.

Figure 2a shows the DSC curves for Pebax^®^Rnew^®^35R53. Here, the melting temperature $T_{m}$ during the first heating phase (blue) is 135.04 °C and 132.82 °C in the second heating phase (red). Furthermore, a glass transition temperature of −22.95 °C was measured in the first heating phase, and −23.66 °C in the second heating phase. The glass transition temperature is superimposed with relaxation effects, as the deflection is not as pronounced [24]. The cooling phase (green) shows a peak at −37.70 °C and 64.10 °C, whereby the latter is attributed to the crystallisation temperature $T_{k}$ of the PA11 segments. This also corresponds to the solidification temperature of the copolymer. The small peaks at 47.69 °C and 83.51 °C are absent in the second heating phase and were attributed to issues with the production of the granules, i.e., rapid cooling. The same problem also influences the $T_{g}$ and $T_{m}$, whereby the values slightly differ from first to second run of the experiment.

Figure 2b presents DSC curves for Pebax^®^Rnew^®^1100. Here, the melting temperature during the first heating phase is 180.83 °C, and 180.24 °C in the second heating phase. An additional deflection was measured at 35.96 °C during the first heating phase, which was attributed to the glass transition temperature. In the second heating phase, a slight change in the melting peak is visible, which is attributed to polymorphic material behaviour.

Figure 2c shows the second heating ramp of both grades. Compared to pure PA11 ($T_{m}$ = 188 °C, $T_{g}$ = 42 °C) [25], the $T_{m}$ values for Pebax^®^Rnew^®^35R53 and Pebax^®^Rnew^®^1100 are slightly lower (see Table 3). Furthermore, it is clearly visible that the $T_{m}$ of Pebax^®^Rnew^®^1100 is higher than that of Pebax^®^Rnew^®^35R53. The increased $T_{m}$ results from the higher proportion of PA11. The melting temperature from the measurements also corresponds to the information on the material data sheets of Arkema [16,23].

### 2.2. Thermo-Gravimetric Analysis (TGA) 

The Pebax grades were subjected to thermo-gravimetric analysis (TGA) using a TGA Q500 from TA instruments (New Castle, Delaware, USA). Three samples with weights between 8 and 12 mg were heated with a constant heat rate of 10 °C/min from 20 to 700 °C in a nitrogen atmosphere and from 700 to 900 °C in the air to analyse thermal degradation.

**Figure 3:**
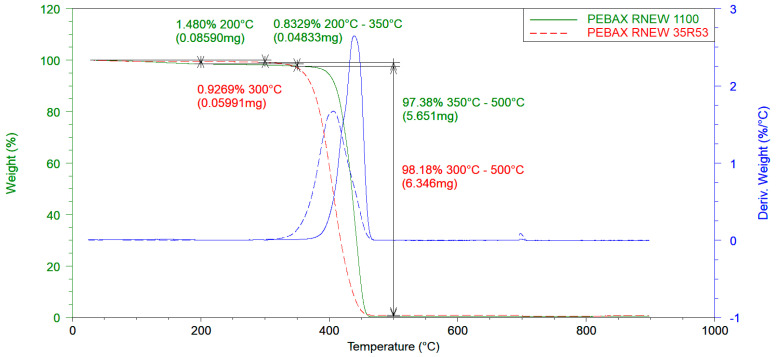
Thermo-gravimetric analysis (TGA) for thermal degradation of Pebax^®^Rnew^®^35R53 and Pebax^®^Rnew^®^1100.

Pebax^®^Rnew^®^35R53 is thermally stable up to a temperature of approximately 300 °C with a mass loss of 0.927 wt.% (see Figure 3). The mass loss also contains the absorbed water. From 300 °C to 700 °C, the polymer decomposes entirely, identified by the noteworthy change in weight. The maximum decomposition of the sample takes place at a temperature of 406 °C, whereby the first derivative shows a distinct peak (blue dotted line).

Pebax^®^Rnew^®^1100 is thermally stable up to a temperature of approximately 350 °C with a mass loss of 2.313 wt.% (see Figure 3). Again, the water stored within the PA molecular chains is mainly responsible for the mass loss up to 350 °C. The accounted mass loss from water evaporation up to 200 °C is 1.480 wt.%. The proportion of stored water in Pebax^®^Rnew^®^1100 is higher compared to Pebax^®^Rnew^®^35R53 which is explained by the higher ratio of PA to polyether blocks of Pebax^®^Rnew^®^1100. From 350 °C to 500 °C, Pebax^®^Rnew^®^1100 decomposes almost completely, indicated by the significant change in weight (peak of blue line). The decomposition temperature (derivative peak at 439 °C) of Pebax^®^Rnew^®^1100 was higher compared with Pebax^®^Rnew^®^35R3.

**Figure 4:**
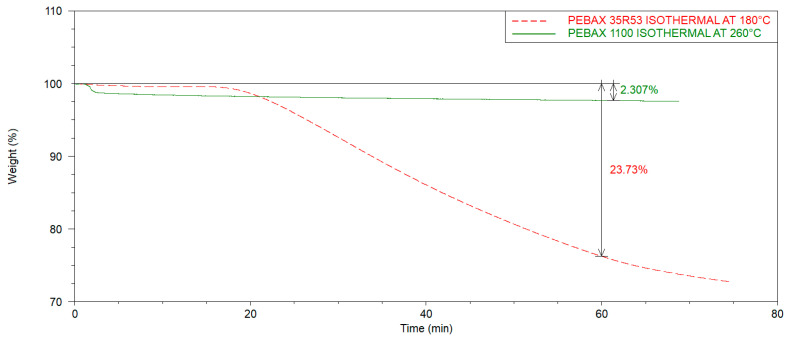
Thermo-gravimetric analysis (TGA) for time-dependent thermal degradation of Pebax^®^Rnew^®^35R53 and Pebax^®^Rnew^®^1100.

Furthermore, isothermal TGA experiments were performed to understand the time-dependent thermal degradation. The samples were heated with a heating ramp of 10 °C/min up to a processing temperature relevant to the respective polymer (Pebax^®^Rnew^®^35R53: 180 °C, Pebax^®^Rnew^®^1100: 260 °C). Subsequently, the temperature remained constant for about one hour. Figure 4 shows the representative curves of both grades. The results of these measurements confirm that especially Pebax^®^Rnew^®^ polymers with a high polyether content are more susceptible to thermal degradation at longer residence times in the processing temperature range.

### 2.3. Analysis of the Temperature-Dependent Flow Behaviour through Rheometric Studies

In the molten state, Pebax^®^Rnew^®^ plastics have a shear thinning behaviour. The increase in the shear rate within the melt consequently causes a decrease in viscosity. The viscosity is essential in filament production for FFF printing as the molten polymer is exposed to shear when pressed through the nozzle. The shear rate depends on the nozzle geometry and the mass flow. The shear rate increases linearly with increasing print speed at different nozzle diameters [26,27]. The relatively low shear rates for filament production and FFF printing range from 0 s^−1^ to 400 s^−1^ [17].

**Figure 5:**
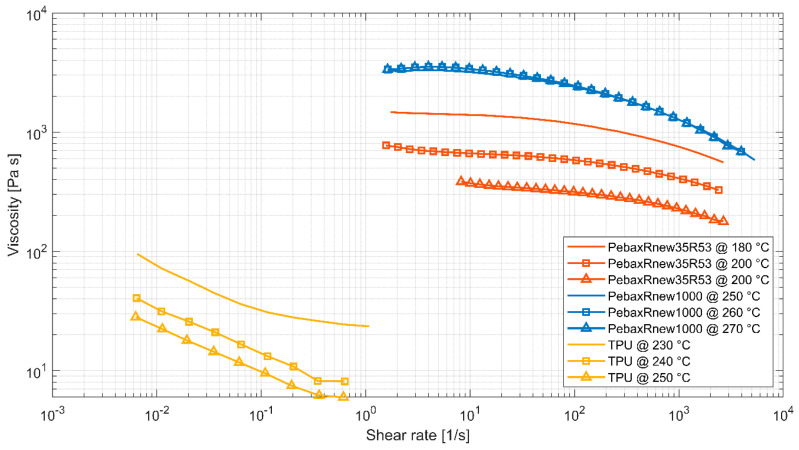
Viscosity behaviour was measured with a plate-to-plate rheometer for grades Pebax^®^Rnew^®^35R53, Pebax^®^Rnew^®^1100, and TPU (Ultimaker TPU 95A [22]).

The temperature-dependent viscosity was determined using a plate-to-plate setup with an MCR 300 rheometer from Physica/Anton Paar (Graz, Austria). A plate diameter of 25 mm with a gap of 1 mm and temperatures of 180, 200, and 220 °C for Pebax^®^Rnew^®^35R53 respectively, 250, 260, and 270 °C for Pebax^®^Rnew^®^1100 were applied (see Figure 5). An excitation of 0.1 to 600 rad/s for the two types of Pebax and 0.01 to 1 rad/s for a standard TPU with a shore hardness of 95A [22] was used to determine the viscosity. The measurements for Pebax^®^Rnew^®^1100 were performed in a nitrogen atmosphere to reduce accelerated thermal degradation effects at higher temperatures. Figure 5 shows the resulting viscosity of Pebax^®^Rnew^®^35R53, Pebax^®^Rnew^®^1100, and TPU (Ultimaker TPU 95A [22]). Here, Pebax^®^Rnew^®^35R53 shows a distinct Newtonian plateau without a strong shear thinning effect resulting in a reduced viscosity. The magnitude of viscosity ranges from 200 to 1200 Pas.

Next, the measurements of Pebax^®^Rnew^®^1100 show deviations due to ongoing thermal degradation, whereby the beginning of the viscosities (zero viscosity) was biased. However, the course of the viscosity is reasonable and shows a more pronounced structural viscosity compared to Pebax^®^Rnew^®^35R53.

The results of the rheological investigations with Pebax^®^Rnew^®^35R53 are reasonable compared to the manufacturer information. The deviation of the results with Pebax^®^Rnew^®^1100 was clarified by performing time-dependent measurements of the zero viscosity. Here, it was shown that despite the inert gas atmosphere of nitrogen, degradation of the surface layer takes place. Nevertheless, the viscosity curve of Pebax^®^Rnew^®^1100 and the results of the frequency-dependent measurements are considered meaningful. Thereof, the results show that the viscosity behaviour of Pebax^®^Rnew^®^35R53 and Pebax^®^Rnew^®^1100 is above the critical shear rate—the so-called shear dilution. However, compared to thermoplastic polyurethane (TPU), shear dilution is less pronounced, as shown in Figure 5.

The results of the rheological measurements of Pebax^®^Rnew^®^35R53 and Pebax^®^Rnew^®^1100 show a shear thinning effect which is smaller compared to a standard TPU, and this will have a positive impact on process stability during FFF processing. 

## 3. Filament Production

**Figure 6:**
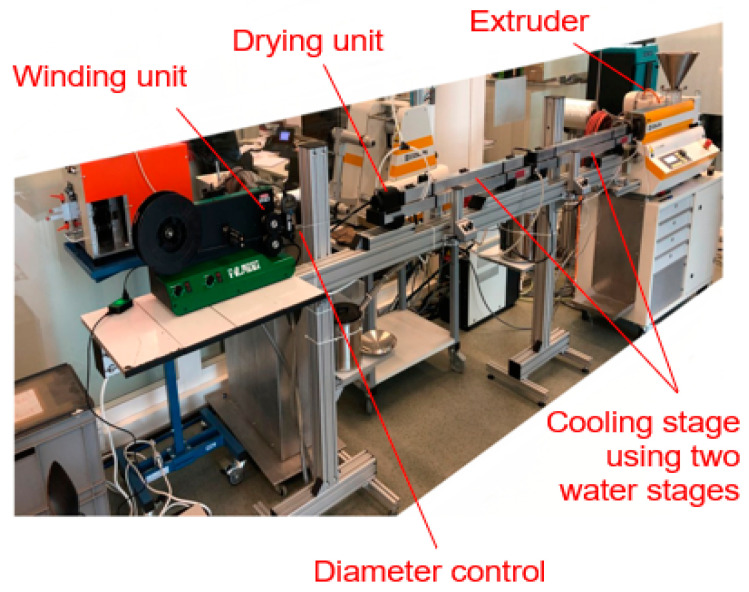
Filament extrusion line at Institute of Polymer Engineering, FHNW University of Applied Sciences and Arts, Northwestern Switzerland.

The filament production was carried out on an extrusion line using a Collin Teach line extruder (Collin, Maitenbeth, Germany), a cooling stage using two water reservoirs, a drying unit, a diameter control (Mitutoyo/Filalogger), and a winding unit from Filabot (Filabot, Barre, VT, USA), see Figure 6. The nozzle diameter was 2 mm, obtaining a filament diameter of 1.75 mm.

**Figure 7:**
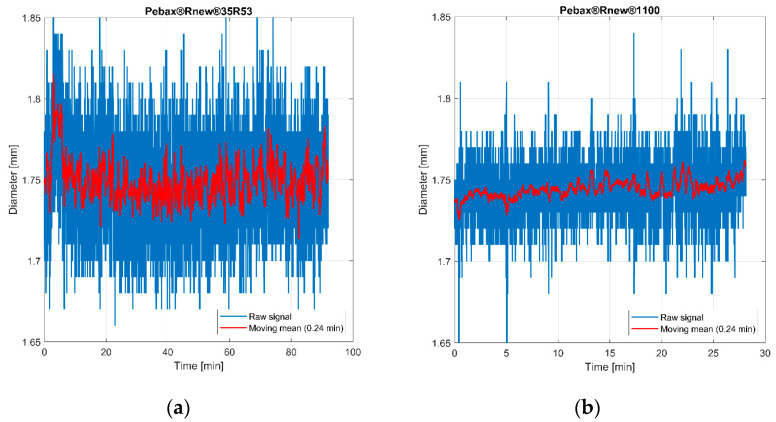
Measurement of filament diameter and shape of the produced filament: (**a**) Pebax^®^Rnew^®^35R53; (**b**) Pebax^®^Rnew^®^1100.

A constant filament diameter of 1.75 +/− 0.05 mm was aimed during filament production. A digital dial gauge (Mitutoyo ID-S1012S, Urdorf, Switzerland) was used to monitor the filament diameter. The measurement results were transferred to the computer software “Filabot” for evaluation. Figure 7 shows the filament diameter during Pebax^®^Rnew^®^35R53 and Pebax^®^Rnew^®^1100 filament production. Here, the blue lines represent all measurement points recorded by the Filabot software. The measurement points were recorded at time intervals of 0.1 s. Therefore, the red line corresponds to the moving average over 144 measurement points each (0.24 min). This line illustrates the trend of the filament diameter during the manufacturing process.

Table 4 summarises the used parameters for the filament extrusion of Pebax^®^Rnew^®^ 35R53 applying temperatures from 190 °C in the extruder, extruder speed of 60 U/min, and a pressure of 15 bar. For Pebax^®^Rnew^®^35R53 filament production, the mean value of all measuring points is 1.749 mm with a standard deviation of 0.024 mm. The extrusion process was stable with an output rate of 1.36 kg/h, a pulling speed of 156 mm/s, and an applied shear rate of 300 s^−1^, and the targeted diameter was achieved. The filament quality check consisted of a qualitative examination of the filament. During the assessment, no pores or foreign particle contamination were found in the Pebax^®^Rnew^®^35R53 filament. 

Table 4 summarises the used parameters for the filament extrusion of Pebax^®^Rnew^®^1100 applying temperatures from 250 °C in the extruder, an extruder speed of 19.2 U/min, and a pressure of 70 bar. The granulate was pre-dried (4–6 h at 75 °C) before filament production due to the negative influence of water uptake on the filament quality. Again, a constant filament diameter of 1.75 +/− 0.05 mm was aimed. Figure 7b shows the filament diameter during Pebax^®^Rnew^®^1100 filament production. The mean value of all measuring points is 1.745 mm with a standard deviation of 0.005 mm. The filament quality check consisted of a qualitative examination of the filament, whereby no pores or foreign particle contamination were found.

A direct comparison of the measured diameter results shows that the Pebax^®^Rnew^®^35R53 has a more significant deviation of the results compared to Pebax^®^Rnew^®^1100. The filament of the Pebax^®^Rnew^®^35R53 started to vibrate within the drying unit, presumably leading to a slight distortion/scattering of the results.

## 4. Specimen Manufacturing and Characterisation

There are different testing standards for tensile strength testing depending on the material. In most cases, the general standard DIN EN ISO 527-1 [28] is used to determine the tensile properties of polymers, according to manufacturers of 3D printing filaments (FFF). However, there is the ISO 37 standard for determining the tensile properties of TPE and elastomers. The results of the different standards are only comparable to a limited extent due to the different test parameters and specimens. For example, the test speed dramatically influences the tensile test results. The strength values and the specimen strains increase with increasing test speed for materials with high strain rates. The DIN EN ISO 527-1 standard was used for all tensile tests to ensure the comparability of results. Table 5 shows a comparison between these two standards. The Youngs modulus for both materials was evaluated by the method described in the standard DIN EN ISO 527-1 [28]. 

In FFF, a thermoplastic in the form of a filament is pressed from a conveying unit into a heated nozzle, making it flowable. Then, the polymer melt is deposited at a previously defined location, creating a three-dimensional geometry layer by layer. This process results in strong anisotropy in all properties. Many influences characterise the mechanical properties of the component. The effects are divided into the following three categories:

- Structure parameters: infill, construction orientation, grid orientation, layer height, layer width, air gap, and part geometry;
- Manufacturing parameters: temperature gradient, nozzle temperature, printing speed, nozzle diameter, and humidity;
- Material: material properties.

The critical factor for the binding force is the possibility of diffusion occurring within the discarded strands. Diffusion between adjacent strands within the same layer leads to better results because the strands were deposited one after the other. Consequently, the temperature and diffusion (trans-layer bindings) are higher. In the case of adjacent strands from different layers, the diffusion is worse because the strands were not deposited one after the other. Thus, the temperature is lower, as well as the diffusion (interlayer bindings) [8,29]. Therefore, it is essential to aim for high bonding strengths between the strands of different layers and the connection between the strands of the same layer or the strand itself. Thus, the tensile strength is lowest when a strand is applied orthogonally to the printing level. The tensile test specimens oriented in angular and flat construction show the highest tensile and bending stiffnesses [30].

**Figure 8:**
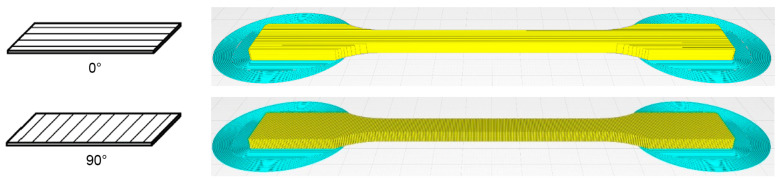
Printed tensile bars with coordinate system (**left**) and the related visual quality (**right**), black arrow shows printing direction.

A Prusa i3 MK3S+ FFF printer (Prusa, Prague, Czech) with a nozzle diameter of 0.6 mm was used for all printing investigations. For Pebax^®^Rnew^®^35R53, five tensile samples with 0° and 90° grid orientations were produced. For the Pebax^®^Rnew^®^1100 filament, five tensile specimens with 0°, 45/−45° and 90° orientations were produced. Figure 8 shows the tensile test rods with 0° and 90° orientations to investigate the anisotropic effect of FFF printed components (see Figure 8). The samples were tested with a Zwick 100 kN tensile test machine (Ulm, Germany) according to standard DIN EN ISO 527-1. The blue areas in Figure 8 are for increased adhesion of the tensile test bar to the print bed. Hence, the additional adhesive area eliminates the need for adhesives/adhesion promoters and prevents warping during the FFF printing process. It was possible to remove the extra adhesive surface for Pebax^®^Rnew^®^35R53 due to the high adhesion of the material to the polyetherimide print bed surface. The FFF printing parameters are shown in Table 6.

**Figure 9:**
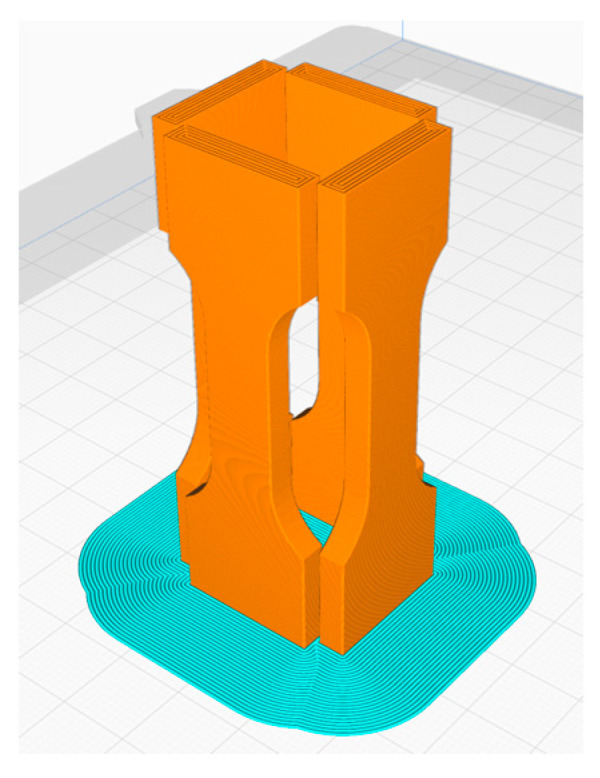
Manufacturing strategy for the tensile test bars ISO 527-1 × 20 mm in Z-direction.

For printing the tensile specimens in the Z-direction, the short tensile specimens were used and arranged in a square to stabilise each other (see Figure 9). Furthermore, an additional adhesive surface was created for the first layer, which can be seen in blue in Figure 9. The FFF printing parameters are shown in Table 7.

**Figure 10:**
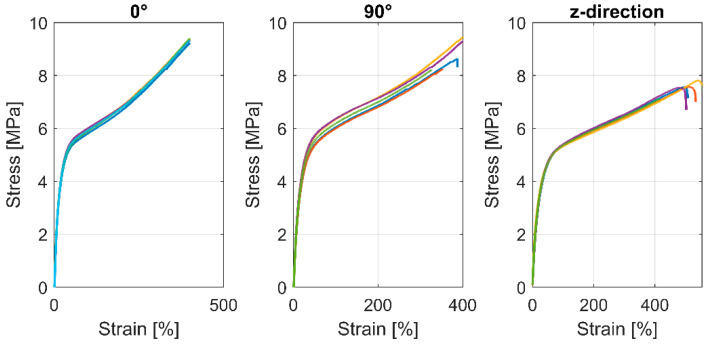
Tensile test results according to DIN standard ISO 527-1 of printed samples with Pebax^®^Rnew^®^35R53.

The samples printed with Pebax^®^Rnew^®^35R53 showed a very flexible and ductile material behaviour (see Figure 10, the different colours represent the samples; in total, 5 per series). The results of tensile tests with Pebax^®^Rnew^®^35R53 are summarised in Table 8. The samples with the standard tensile test rod geometry according to ISO 527-1 with 0° and 90° orientation showed no significant differences. However, the results of these tensile tests, particularly the modulus of elasticity, are only of limited significance, as the determination of the modulus of elasticity with flexible plastics is difficult. In the tensile testing of metals, there is a large and distinct range in which Hooke’s law applies, whereby a direct proportionality between stress and strain occurs. For elastomers and very flexible TPE polymers, there is only a minimal area in which Hook’s law applies. Such materials are almost always used in areas where no single modulus of elasticity can be specified. In tensile tests, it is often customary to determine the stress values, e.g., at 100% or 200% strain. No failure was measured in the case of 0° because the machine’s capacity with 400% strain was reached.

**Figure 11:**
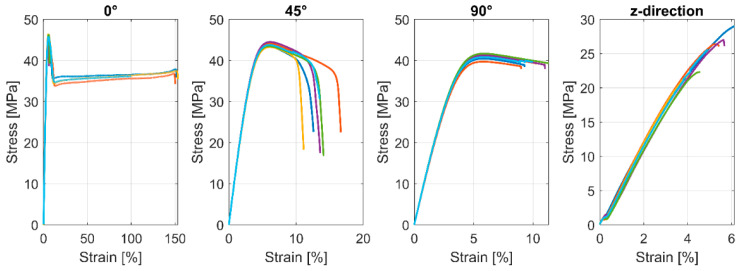
Tensile test results according to DIN standard ISO 527-1 of printed samples with Pebax^®^Rnew^®^1100.

The results of the Pebax^®^Rnew^®^1100 specimens with the standard tensile bar geometry according to ISO 527-1 with 0°, 90°, and 45°/−45° grid orientation showed no significant differences in terms of Young’s modulus and elastic behaviour. However, the plastic behaviour varies significantly depending on the grid orientation, particularly illustrated by the elongation of Pebax^®^Rnew^®^1100 with 0° grid orientation in Figure 11 (the different colours represent the samples; in total, 5 per series). The 45°/−45° FFF-3D printed tensile test bars cannot be used to determine the shear modulus since no significant differences in the results of Pebax^®^Rnew^®^1100 specimens with 45°/−45° grid orientations were found. The standard ASTM D3518-1 for composites uses tensile test bars with 45°/−45° fibre orientation to determine the shear modulus.

The curves of the tensile test bars printed in the Z-direction show brittle behaviour in the stress–strain diagram. Table 9 shows that the Young’s modulus of the tensile test bars printed in the Z-direction is much lower than that of the specimens with 0°, 90°, and 45°/−45° grid orientation. The mechanical properties of the Pebax^®^Rnew^®^1100 specimens revealed a transversely isotropic material behaviour because the X and Y directions of the FFF printed parts have the same elastic range, and only the tensile specimens printed in the z-direction show a different behaviour.

In general, tensile tests with Pebax^®^Rnew^®^35R53 and Pebax^®^Rnew^®^1100 show the difference between a flexible and a rigid polymer. However, there were slight deviations in both the test parameters and the tensile rod geometry, which eventually led to more minor deviations in the mechanical properties (see Table 10). For the more flexible Pebax^®^Rnew^®^35R53 material, it should be mentioned that the tensile test according to ISO 527-1 is only partially suitable—particularly for determining the Young’s Modulus.

### Multi-Material Tensile Testing

Additionally, investigations with multi-material FFF printing of Pebax^®^Rnew^®^35R53 and Pebax^®^Rnew^®^1100 were performed. Here, five test specimens with the same parameters as in Table 7 were used. For the multi-material tensile tests, the mechanical properties and load cases relevant to the two-component (2K) injection moulding were used as a basis. Here, the adhesion between the two material components is a decisive factor for the overall quality and resilience. However, there is no standardised procedure for testing adhesion in 2K injection moulding. Therefore, the multi-material tensile tests for elastomers were tested according to ISO 527. As a measure of the quality of the composite, the maximum stress and the elongation at the break of the connection were analysed. Hard/soft connections are often also exposed to peeling stresses, which are usually more critical than pure tensile loads [31]. Within the scope of this study, only multi-material tensile tests were carried out as a simplified test scenario. Consequently, peeling stresses or 3-point bending tests were neglected.

**Figure 12:**
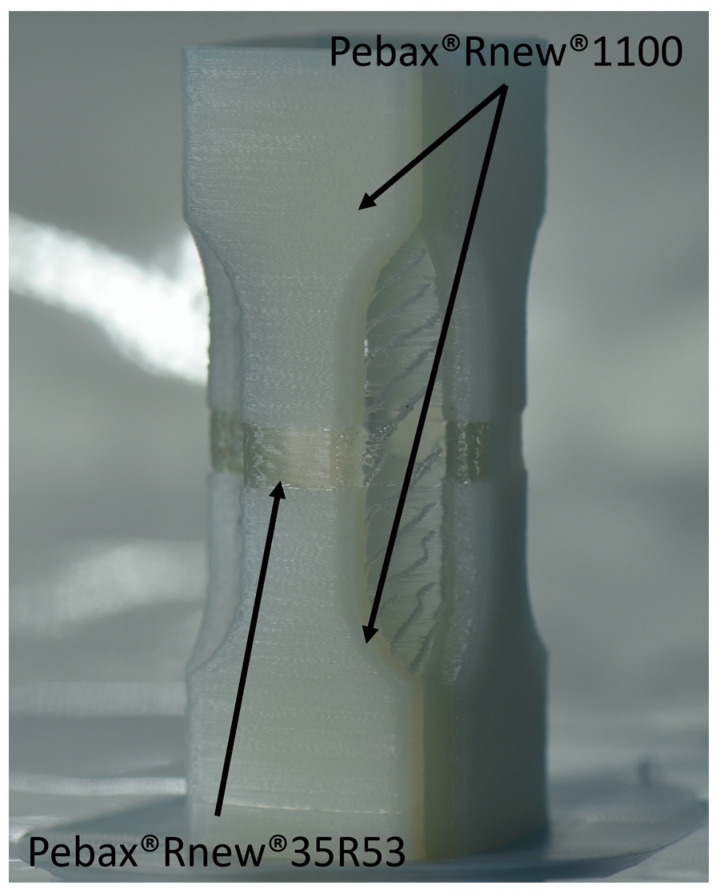
Multi-material tensile test bar after the finished FFF printing process.

With a three-axis FFF printer without automated filament change, it is only possible to produce multi-material tension test rods in the z-direction. Here, the shorter test specimens ISO 527-1 were used. For mutual stabilisation, the multi-material tensile specimens were arranged in a square for production (see Figure 12). Pebax^®^Rnew^®^1100 filament was used as the base material. A 5 mm broad segment of Pebax^®^Rnew^®^35R53 was printed in the middle of the tension test rod. Figure 12 shows the multi-material tensile bars.

**Figure 13:**
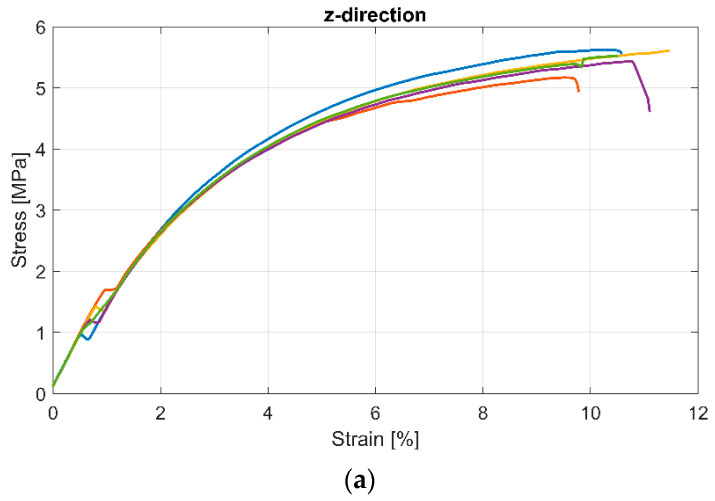

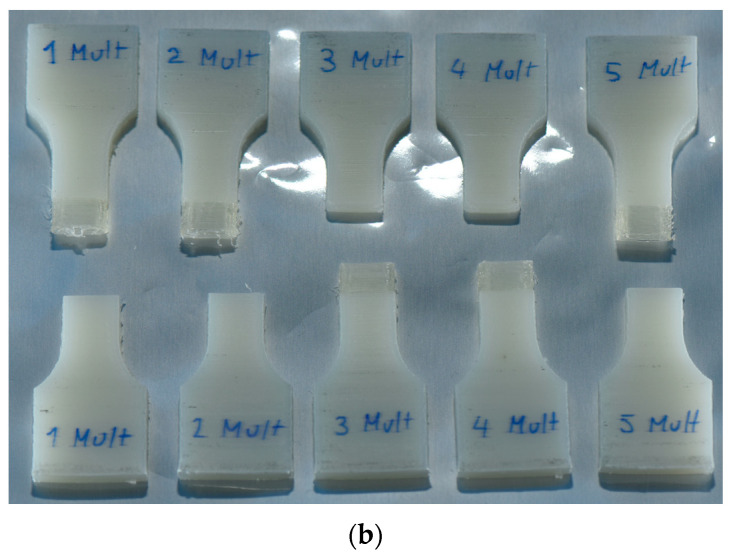
Mechanical characterisation of multi-material tensile tests: (**a**) Stress–strain diagrams of the multi-material tensile tests with Pebax^®^Rnew^®^35R53 and Pebax^®^Rnew^®^1100; (**b**) Fracture analysis of tested multi-material tensile tests with Pebax^®^Rnew^®^35R53 and Pebax^®^Rnew^®^1100.

Figure 13 shows the mechanical characterisation of the multi-material test specimens (the different colours represent the samples; in total, 5 per series). The sample does not show a pronounced plastic behaviour in the stress–strain diagram (Figure 13a). Additionally, no plastic deformations were visible from the bare eye on the fractured samples (Figure 13b). Hence, the broken area showed a dominant cohesive failure between the interface of the two grades. 

In the multi-material tensile specimens with Pebax^®^Rnew^®^35R53 and Pebax^®^Rnew^®^1100, an adhesion fracture occurs, which occurs at maximum stress of 5.5 ± 0.18 MPa and an elongation at break of 10.5 ± 0.71%.

The value for the maximum stress of 5.5 ± 0.18 MPa of the multi-material tensile specimens is surprisingly high compared to the maximum stress of 6.7 ± 0.95 MPa of the Pebax^®^Rnew^®^35R53 tensile specimens.

## 5. Conclusions

In this study, for the first time, the bio-based materials Pebax^®^Rnew^®^35R53 and Pebax^®^Rnew^®^1100, with their excellent chemical and physical properties, were investigated for their potential for FFF printing. It was found that both material types are applicable in FFF, and related mechanical parameters were evaluated. 

The material characterisation has shown that both Pebax^®^Rnew^®^35R53 and Pebax^®^Rnew^®^1100 have reasonable processing properties with a thermal stability of over 300 °C. The decomposition temperature derived via thermogravimetric analysis for Pebax^®^Rnew^®^35R53 is 406 °C and for Pebax^®^Rnew^®^1100 is 439 °C. Therefore, the processing temperatures required for Pebax^®^Rnew^®^35R53 (max. 205 °C for filament production, 230 °C for FFF) and Pebax^®^Rnew^®^1100 (max. 245 °C for filament production, 270 °C for FFF) do not lead to thermal degradation. However, it must be considered that both grades, especially Pebax^®^Rnew^®^35R53, are prone to thermal degradation during long residence times in the molten state. The manufactured filament showed favourable tolerances (Pebax^®^Rnew^®^35R53: 1.749 ± 0.024 mm, Pebax^®^Rnew^®^1100: 1.745 ± 0.005 mm) suitable for FFF applications. The FFF printing parameters were experimentally determined:

- Pebax^®^Rnew^®^35R53: nozzle temperature 220–230 °C, print bed 50 °C (Table 6 and Table 7);
- Pebax^®^Rnew^®^1100: nozzle temperature 250–270 °C, print bed 50 °C (Table 6 and Table 7).

In summary, the performed study shows that a bio-based Pebax is suitable for FFF processing with improved process stability due to less shear thinning effect and good mechanical performance compared to a standard flexible material (TPU [22]). Combining the two materials in one design is a promising concept as the adhesion strength is 5.5 ± 0.18 MPa, which is the strength in the Z-direction of the flexible Pebax^®^Rnew^®^35R53 grade. This study is a starting point for further studies to optimise processing parameters and to achieve higher mechanical parameters.

We want to close this conclusion with an outlook for potential applications. Potential interesting applications are in metamaterial structures. Metamaterials are artificial structures or composites with exceptional physical properties that do not occur in natural materials. Metamaterial structures open new design and application possibilities for various components. The dimensioning/construction of metamaterial structures requires a different mindset than conventional materials. The adaptation of the required material properties is carried out by the correct selection and dimensioning of the metamaterial structure and not by a modification of the chemical components of the material. Through proper selection and dimensioning of a metamaterial structure, for example, a shoe sole cushioning can be adapted individually to each person. Hybrid designs can be enabled with materials available as soft and rigid polymer grades.

## Figures and Tables

**Table 1:** Comparison of Pebax^®^Rnew^®^35R53 filaments with different materials available on the market for fused filament fabrication (FFF).

Material	References	Manufacturer	Shore-Hardness	Young’s Modulus	Density
Pebax^®^Rnew^®^35R53	[16]	Arkema (Colombes, France)	25 D	40 MPa	1.02 g/cm^3^
70A Filaflex Ultra-Soft	[17]	Recreus (Elda, Spain)	22 D/70 A	32 MPa	1.08 g/cm^3^
FiberFlex 30D	[18]	Fiberlogy (Brzezie, Poland)	30 D	-	1.07 g/cm^3^
Filaflex Purifier	[19]	Recreus (Elda, Spain)	31 D	20 MPa	1.17 g/cm^3^
Arnitel^®^ ID 2045 TPC	[20]	Formfutura (Nijmegen, Netherlands)	34 D	29 MPa	1.10 g/cm^3^
Kimya TPU-92A	[21]	Kimya (Nantes, France)	40 D/92 A	90 MPa	1.16 g/cm^3^
TPU 95A	[22]	Ultimaker (Utrecht, Netherlands)	95 A	26 MPa	1.22 g/cm^3^

**Table 2:** Comparison of Pebax^®^Rnew^®^35R53 to Pebax^®^Rnew^®^1100.

Property	Pebax^®^Rnew^®^35R53 [16]	Pebax^®^Rnew^®^1100 [23]
Amount of PA11	29 % (ASTM D6866)	65% (-)
Melting temperature	135 °C (ISO 11357-1/-3)	188 °C (ISO 11357)
Density	1020 kg/m^3^ (ISO 1183)	980 kg/m^3^ (ISO 1183)
Young’s Modulus	40 MPa (ISO 527-1/-2)	1160 MPa (ISO 178)
Shore D Hardness	25 (ISO 7619-1)	68 (ISO 868)

**Table 3:** Comparison of thermal properties from DSC experiments with datasheets from the manufacturer.

Property	Pebax^®^Rnew^®^35R53 [16]	Pebax^®^Rnew^®^1100 [23]	PA11 [25]
$T_{g}$ (datasheet)	-	-	42 °C
$T_{g}$ (DSC)	−23 °C	37.57 °C	-
$T_{m}$ (datasheet)	135 °C	188 °C	188 °C
$T_{m}$ (DSC)	132.83 °C	187.57 °C	-

**Table 4:** Processing parameters for the filament production of Pebax^®^Rnew^®^ 35R53.

Parameter	Pebax^®^Rnew^®^35R53	Pebax^®^Rnew^®^1100
Extruder zone 1 (Feed)	40 °C	40 °C
Extruder zone 2	205 °C	230 °C
Extruder zone 3	200 °C	245 °C
Extruder zone 4	190 °C	250 °C
Extruder zone 7 (nozzle)	190 °C	250 °C
Water reservoir 1	45 °C	35 °C
Water reservoir 2	25 °C	25 °C
Extruder speed	60 U/min	19.2 U/min
Pressure	15 bar	70 bar
Haul-off speed	156 mm/s	163 mm/s
Feed rate	1.36 kg/h	1.42 kg/h

**Table 5:** Comparison of DIN EN ISO 527-1 with ISO 37.

Parameter	ISO 527-1	ISO 37
Specimens	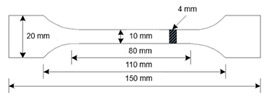	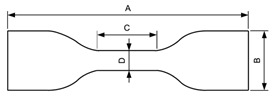
Test speed	1–500 mm/min	200–500 mm/min
Results	Yield stress Breaking stress Tensile strength Elongation at yield Elongation at break (Tensile) modulus of elasticity	Tensile strength at break Elongation at break Tensile stress at 100% elongation Tensile stress at 200% elongation Tensile stress at 300% elongation

**Table 6:** FFF-3D printing parameters of tensile test bars with 0° and 90° orientation in XY-direction.

Parameter	Pebax^®^Rnew^®^35R53	Pebax^®^Rnew^®^1100
Layer height	0.15 mm	0.15 mm
Line width (0.6 Nozzle)	0.55 mm	0.55 mm
Material flow	100%	100%
Nozzle temperature	230 °C	260 °C
Print bed temperature	50 °C	100 °C
Print speed	20 mm/s	25 mm/s
Cooling	0%	0%

**Table 7:** FFF-3D printing parameters of the tensile test bars in the Z-direction.

Parameter	Pebax^®^Rnew^®^35R53	Pebax^®^Rnew^®^1100
Layer height	0.15 mm	0.15 mm
Line width (0.6 Nozzle)	0.55 mm	0.55 mm
Material flow	100%	100%
Nozzle temperature	230 °C	270 °C
Print bed temperature	50 °C	100 °C
Print speed	15 mm/s	15 mm/s
Cooling	100%	100%
Retraction	0.85 mm	3 mm

**Table 8:** Results of tensile tests with Pebax^®^Rnew^®^35R53.

Direction	Young’s Modulus $E_{t}$	Tensile Stress at 100% Elongation $\sigma_{100\%}$	Tensile Stress at 200% Elongation $\sigma_{200\%}\;$	Tensile Strength at Break $\sigma_{m}$
0°	3.83 ± 1.6 MPa	5.93 ± 0.1 MPa	6.79 ± 0.1 MPa	9.61 ± 0.1 MPa
90°	6.49 ± 5.8 MPa	6.19 ± 0.2 MPa	7.00 ± 0.2 MPa	7.98 ± 2.2 MPa
Z-direction	16.12 ± 3.2 MPa	5.36 ± 0.0 MPa	5.94 ± 0.1 MPa	7.07 ± 1.1 MPa

**Table 9:** Results of tensile tests with Pebax^®^Rnew^®^1100.

Direction	Young’s Modulus $E_{t}$	Tensile Strength Max *σ_m_*	Max Strain at Failure ε_m_
0°	1287.74 ± 13.9 MPa	45.85 ± 0.6 MPa	5.67 ± 0.1%
90°	1215.34 ± 11.8 MPa	40.83 ± 0.7 MPa	5.83 ± 0.15%
45°/−45°	1270.41 ± 9.8 MPa	43.78 ± 0.5 MPa	6.02 ± 0.1%
Z-direction	322.54 ± 132.8 MPa	25.36 ± 2.7 MPa	5.15 ± 0.7%

**Table 10:** Comparison of the Young’s modulus of the tensile tests with the datasheet.

Direction	Young’s Modulus of Pebax^®^Rnew^®^35R53	Young’s Modulus of Pebax^®^Rnew^®^1100
0°	3.83 ± 1.6 MPa	1287.74 ± 13.9 MPa
90°	6.49 ± 5.8 MPa	1215.34 ± 11.8 MPa
45°/−45°	-	1270.41 ± 9.8 MPa
Z-direction	16.12 ± 3.2 MPa	322.54 ± 132.8 MPa
Data sheet	25 MPa [16]	1160 MPa [23]

## Data Availability

The data presented in this study are available on request from the corresponding author.

## References

[1] Srivastava M., Rathee S. (2022). Additive manufacturing: Recent trends, applications and future outlooks. Prog. Addit. Manuf..

[2] Meier M., Tan K.H., Lim M.K., Chung L. (2019). Unlocking innovation in the sport industry through additive manufacturing. Bus. Process Manag. J..

[3] Jenner F., Dharmani S. How the Future of 3D Printing is Taking Shape. https://www.ey.com/en_gl/advanced-manufacturing/how-the-future-of-3d-printing-is-taking-shape.

[4] Grieder S., Zhilyaev I., Küng M., Brauner C., Akermann M., Bosshard J., Inderkum P., Francisco J., Willemin Y., Eichenhofer M. (2022). Consolidation of Additive Manufactured Continuous Carbon Fiber Reinforced Polyamide 12 Composites and the Development of Process-Related Numerical Simulation Methods. Polymers.

[5] Kurzynowski T., Pawlak A., Smolina I. (2020). The potential of SLM technology for processing magnesium alloys in aerospace industry. Arch. Civ. Mech. Eng..

[6] Auricchio F., Marconi S. (2016). 3D printing: Clinical applications in orthopaedics and traumatology. EFORT Open Rev..

[7] Karevska S., Steinberg G., Müller A., Wienken R., Kilger C., Krauss D. 3D Printing: Hype or Game Changer?. https://assets.ey.com/content/dam/ey-sites/ey-com/en_gl/topics/advisory/ey-3d-printing-game-changer.pdf.

[8] Kamalpreet S., Sunpreet S., Chander P., Karupppasamy S.S.R. (2022). Sustainability for 3D Printing.

[9] Pal A.K., Mohanty A.K., Misra M. (2021). Additive manufacturing technology of polymeric materials for customised products: Recent developments and future prospective. RSC Adv..

[10] Arkema a Very Special Kind of Polymer Chemistry. https://pebaxpowered.arkema.com/en/pebax-technology/.

[11] Lligadas G., Ronda J.C., Galià M., Cádiz V. (2013). Renewable polymeric materials from vegetable oils: A perspective. Mater. Today.

[12] Koh M.H., Kim H., Shin N., Kim H.S., Yoo D., Kim Y.G. (2012). Divergent process for C 10, C 11 and C 12 ω-amino acid and α,ω-dicarboxylic acid monomers of polyamides from castor oil as a renewable resource. Bull. Korean Chem. Soc..

[13] Tuhin M.O., Ryan J.J., Sadler J.D., Han Z., Lee B., Smith S.D., Pasquinelli M.A., Spontak R.J. (2018). Microphase-Separated Morphologies and Molecular Network Topologies in Multiblock Copolymer Gels. Macromolecules.

[14] Bates F.S. (1991). Polymer-polymer phase behavior. Science.

[15] Car A., Drioli E., Giorno L. (2014). Polyether Block Amide (PEBAX). Encyclopedia of Membranes.

[16] Datasheet Pebax® Rnew® 35R53 SP 01. https://www.materialdatacenter.com/ms/en/Pebax/Arkema/Pebax%C2%AE+Rnew%C2%AE+35R53+SP+01/2e6a1621/264.

[17] Filaflex 70A. https://recreus.com/de/filamente/6-filaflex-70a.html.

[18] FIBERFLEX 30D. https://fiberlogy.com/de/filamente/fiberflex-30d/.

[19] Filaflex Purifier. https://recreus.com/de/filamente/14-filaflex-purifier.html.

[20] Arnitel® ID 2045 Natural. https://www.3djake.ch/de-CH/formfutura/arnitelr-id-2045-natural.

[21] Kimya TPU-92A. https://www.kimya.fr/de/produkt/tpu-92a-kimya-3d-filament/.

[22] TPU 95A. https://ultimaker.com/de/materials/tpu-95a.

[23] Datasheet PEBAX^®^RNEW^®^1100. https://www.arkema.com/global/en/products/product-finder/product-range/technicalpolymers/pebax-product-family/.

[24] Kaiser W. (2021). Kunststoffchemie für Ingenieure.

[25] Mark J.E. (2009). Polymer Data Handbook.

[26] Brauner C., Küng M., Arslan D., Maurer C. (2021). Fused filament fabrication based on polyhydroxy ether (Phenoxy) polymers and related properties. Polymers.

[27] Cuan-Urquizo E., Barocio E., Tejada-Ortigoza V., Pipes R.B., Rodriguez C.A., Roman-Flores A. (2019). Characterization of the mechanical properties of FFF structures and materials: A review on the experimental, computational and theoretical approaches. Materials.

[28] DIN EN ISO 527-1:2019-12. https://www.beuth.de/de/norm/din-en-iso-527-1/306958894.

[29] Luo C., Mrinal M., Wang X., Hong Y. (2021). Bonding widths of deposited polymer strands in additive manufacturing. Materials.

[30] Floor J., Van Deursen B., Tempelman E. (2018). Tensile strength of 3D printed materials: Review and reassessment of test parameters. Mater. Test..

[31] Ahlers D., Wasserfall F., Hendrich N., Zhang J. 3D printing of nonplanar layers for smooth surface generation. Proceedings of the IEEE International Conference on Automation Science and Engineering.

